# An image dataset of macroscopic appearance of wood formation in Anthocephalus macrophyllus based on the pinning method

**DOI:** 10.1016/j.dib.2024.110187

**Published:** 2024-02-12

**Authors:** Bahidin Laode Mpapa, Imam Wahyudi, Istie Sekartining Rahayu, Andi Detti Yunianti

**Affiliations:** a Forest Product Science and Technology Study Program, SPs IPB University, Bogor, 16680, Indonesia; b Agrotechnology Study Program, Faculty of Agriculture, Muhammadiyah University of Luwuk Banggai, Luwuk, 94711, Indonesia; c Department of Forest Products, Faculty of Forestry and Environment, IPB University, Bogor 16680, Indonesia; d Forest Engineering, Faculty of Forestry, Hasanuddin University, Makassar, 90245, Indonesia

**Keywords:** Wood bark appearance, Formation area, Image analysis, Wound pinning

## Abstract

This article describes a dataset of images depicting the monthly wood formation of Anthocephalus macrophyllus throughout the year. The images were obtained from cross-sectional stem sections of the tree starting from January, February, March, April, May, June, July, August, September, October, November, and December 2021. The image capture process was conducted at the Nano Imaging-Advanced Research Laboratory, IPB University, using the Keyence VHX-7000 digital microscope with a magnification of 20x. Each image was in JPG format with dimensions of 2880 × 2160, providing a comprehensive overview of wood formation each month. The pinning process resulted in distinct marks in both the bark and wood. In the bark, damage and healing due to injuries are clearly visible. Similarly, in the wood section, the wood formation area was clearly evident. The dataset on wood formation based on the application of pinning provides an interesting image analysis for observing wood formation in tropical regions. The data generated from different locations and climates can serve as comparative data for observing wood formation in other species.

Specifications Table

**Table:** 

Subject	Tree growth science, wood quality
Specific subject area	Image dataset for wood formation
Data format	Raw: JPG
Type of data	Image
Data collection	Macroscopic images of the wood formation were captured using a Keyence VHX-7000 digital microscope at a magnification of 20x. The capturing was done at the Nano Imaging-Advanced Research Laboratory, IPB University, Bogor, Indonesia (6°03′16.5" South Latitude and 106°04′25.8" East Longitude). The original image format is JPG, with dimensions of 2880 × 2160 pixels. These images illustrate the monthly wood formation of Anthocephalus macrophyllus throughout 2021. The dataset folder size was 16.2 MB, and a compressed file in the ZIP format was provided for easy download.
Data source location	Wood formation images were obtained at the Nano Imaging-Advanced Research Laboratory, IPB University, Bogor, Indonesia (6°03′16.5’ South Latitude, 106°04′25.8″ East Longitude). The Anthocephalus macrophyllus wood samples were sourced from Bunga Village, North Luwuk District, Banggai Regency, Central Sulawesi Province (0°52′26.23" South Latitude and 122°54′04.19" East Longitude), altitude 304 meters above sea level.
Data accessibility	Repository name: Mendeley Data Data identification number: 10.17632/b6bn3b3szw.1 Direct URL to data: https://data.mendeley.com/datasets/b6bn3b3szw/1

## 1 Value of the Data

• The dataset of macroscopic images of Anthocephalus macrophyllus wood formation after pinning application aids researchers in observing the formation of tropical woods at different locations, types, and climatic conditions. Additionally, it enables precise study of the impact of pinning on the bark and wood over specific periods.
• The image dataset comprises macroscopic representations of wood formation for each month throughout the year. Wood researchers can use this dataset to compare wood formations among different species. Researchers involved in tree quality detection using specific sensors can utilise this dataset to develop digital tools that provide information on wood formation and tree quality at each age level.
• The dataset of Anthocephalus macrophyllus wood formation images was obtained from reliable digital microscope captures, facilitating the provision of accurate information for the identification of fast- and slow-growing species.
• Monthly image datasets can assist researchers in detecting the appearance of damaged and healthy wood tissues during specific periods. Furthermore, it helps calculate the area of wood formation based on the pinning marks.

## 2 Background

The lack of information on tropical wood formation research based on the pinning method is the primary reason for writing this manuscript. Wounding the tree trunk by pinning is closely related to monitoring wood formation events. The pinning method is useful for studying events within wood formation [1]. The concept of pinning is based on the simple experience that tree cambium is highly responsive to external impacts. The wound response due to pinning varies significantly among species, shape and size of the wound, seasons throughout the year, growth rates, and needle sizes [2]. The formed wounds allow monitoring of wood formation over a specific period [3]. The pinning method of wounding has been primarily applied to trees in temperate climate regions [4], [5], [6]. The pinning method for tropical trees involves the use of nails or pins because tropical trees generally have thick and hard bark [7,8].

## 3 Data Description

**Fig. 1:**
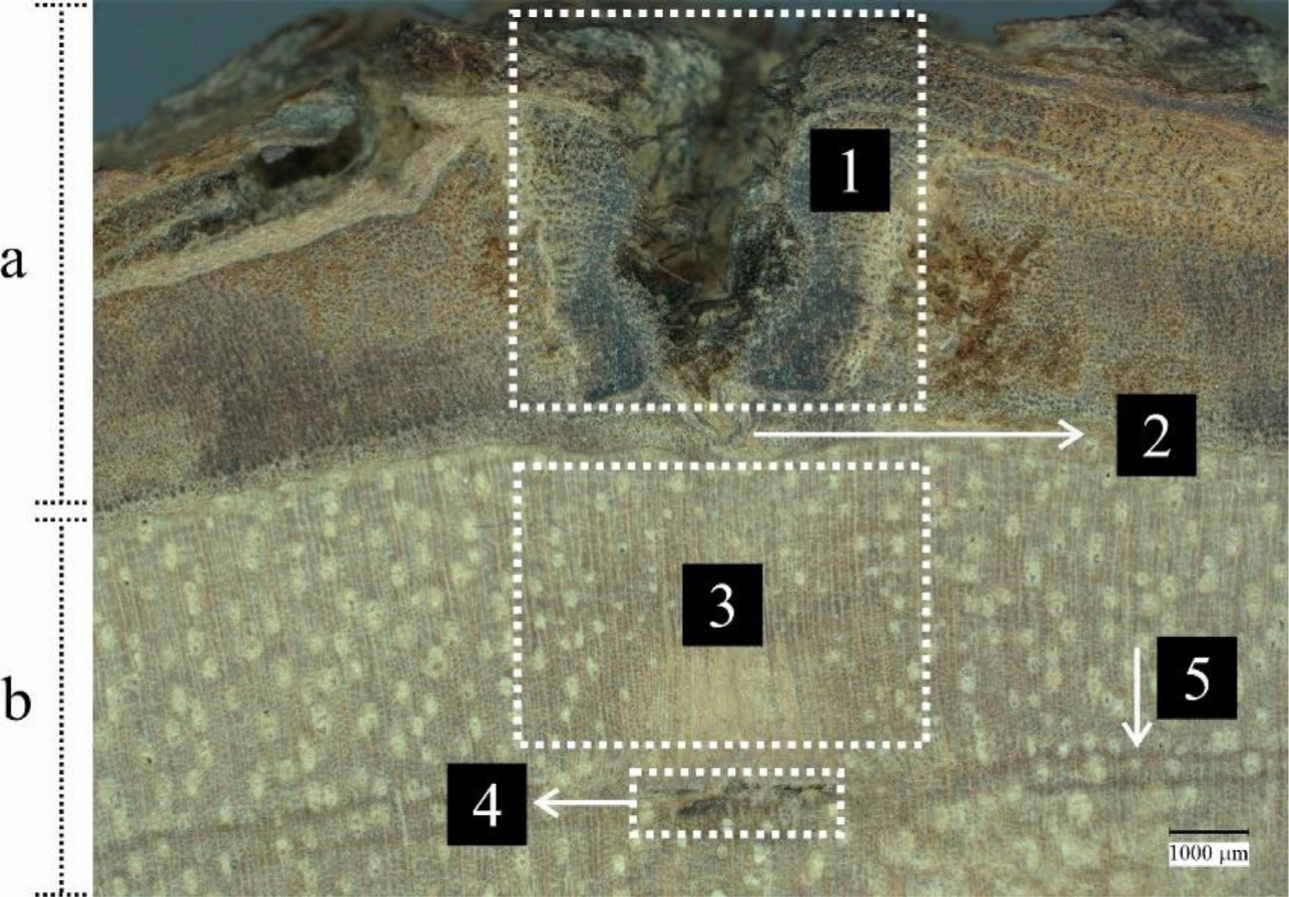
Example section of macroscopic cross-sectional image of Anthocephalus macrophyllus wood. Bark section (a): damaged area owing to pinning (1) and healing area of the bark (2). Wood section (b): wood formation area (3), initial scar from the wound (4), and formed line on the side of the initial wound scar (5).

The dataset described in this article consists of 12 cross-sectional images of Anthocephalus macrophyllus wood, clearly showing wounds caused by pinning in both bark and wood. These images cover the wood formation period from January to December 2021. Image 1 represents wood formation in January (30 days), image 2 in February (60 days), image 3 in March (90 days), image 4 in April (120 days), image 5 in May (150 days), image 6 in June (180 days), image 7 in July (210 days), image 8 in August (240 days), image 9 in September (270 days), image 10 in October (300 days), image 11 in November (330 days), and image 12 in December (360 days). Image capture was conducted meticulously following standard procedures at the Nano Imaging-Advanced Research Laboratory, IPB University, using a Keyence VHX-7000 digital microscope with 20x magnification. All original images were in the JPG format with dimensions of 2880 × 2160 pixels. The image descriptions are shown in Fig. 1.

The entire image dataset from January to December (12 images) was analyzed using ImageJ software to determine the width of wood formation. Table 1 shows the calculation of the width of wood formation for one year for the species Anthocephalus macrophyllus.

**Table 1:** Sample width of wood formation Anthocephalus macrophyllus.

Image dataset	Width of wood formation (µm)
January (30 days)	248.71
February (60 days)	601.04
March (90 days)	1057.00
April (120 days)	1077.72
May (150 days)	1326.43
June (180 days)	2901.55
July (210 days)	3005.18
August (240 days)	3191.71
September (270 days)	3212.44
October (300 days)	3170.98
November (330 days)	3564.77
December (360 days)	4124.35
Total amount	27481.86⁎

⁎ 27481.86 micrometer = 27.48 mm = 2.74 cm.

## 4 Experimental Design, Materials and Methods

### 4.1 Procedure for capturing macroscopic wood formation images

**Fig. 2:**
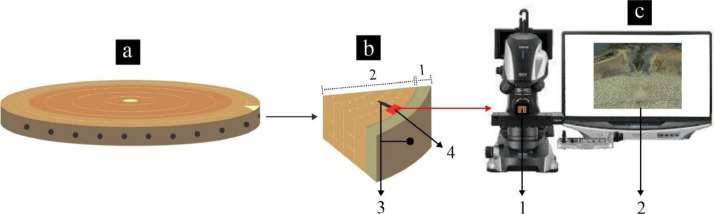
Workflow for image dataset collection. Wood disc at diameter breast height (a), sample section in the pinning area (b): bark (1), wood (2), pinning mark (3), and wood formation area (4). Keyence VHX-7000 digital microscope tool (c): capturing macroscopic images of the wood formation area (1) and the resulting images (2).

Fig. 2 illustrates a series of steps to obtain macroscopic views of Anthocephalus macrophyllus wood formation. It begins by obtaining a wood section in the form of a disc with a thickness of ± 5 cm, precisely in the area affected by pinning. Subsequently, small sections measuring 2 × 2 × 2 cm were created. These sections must be crafted with extreme care, totalling 12 pieces (January to December), particularly focusing on areas with pinning marks. Next, the surface of these sections was sanded to achieve a smooth surface, enhancing the visibility of the wood formation, initial pinning marks, and damage to the bark. Once the sample sections were prepared, they were positioned on a digital microscope stage. The magnification and focus were adjusted, and images were captured one by one.

### 4.2 Workflow measuring the width of wood formation

**Fig. 3:**
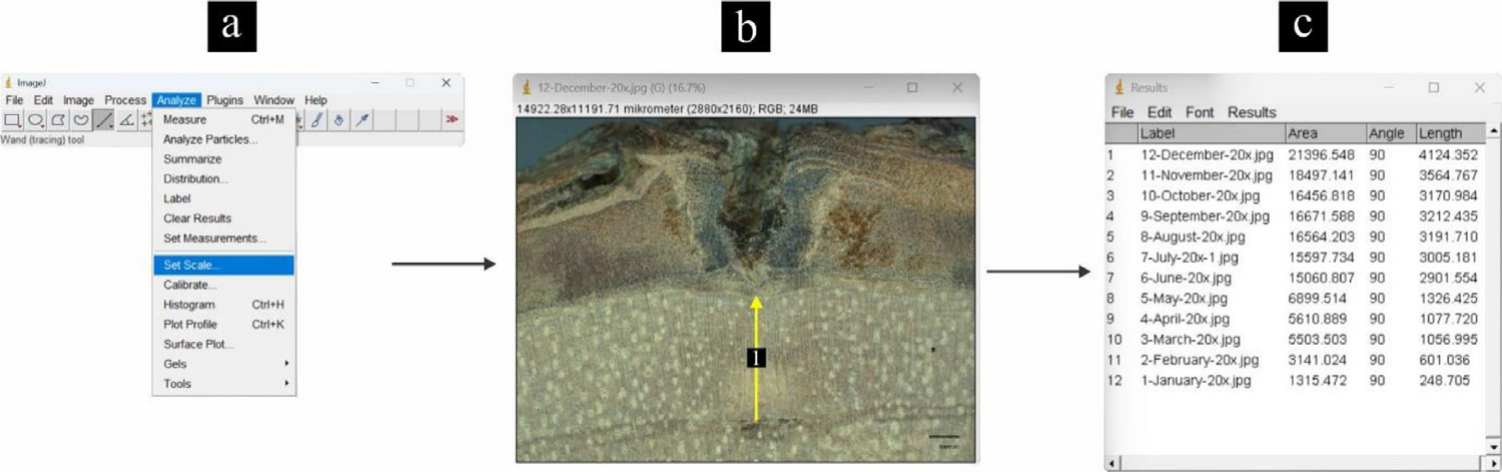
Workflow measuring the width of the wood formation. Software ImageJ (a), measurement of the width of wood formation (b), and measurement results (c).

Fig. 3 shows a series of measurements of the width of wood formation using the ImageJ software. The width of the wood formation was measured from the initial mark of the opening to the inner bark near the cambium. Before taking the measurements, we first enabled and set the scale in the ImageJ software to match the scale in the image to be measured. A one-by-one image dataset from January to December was input to the ImageJ software and measurements were made.

### 4.3 Dataset image management

**Fig. 4:**
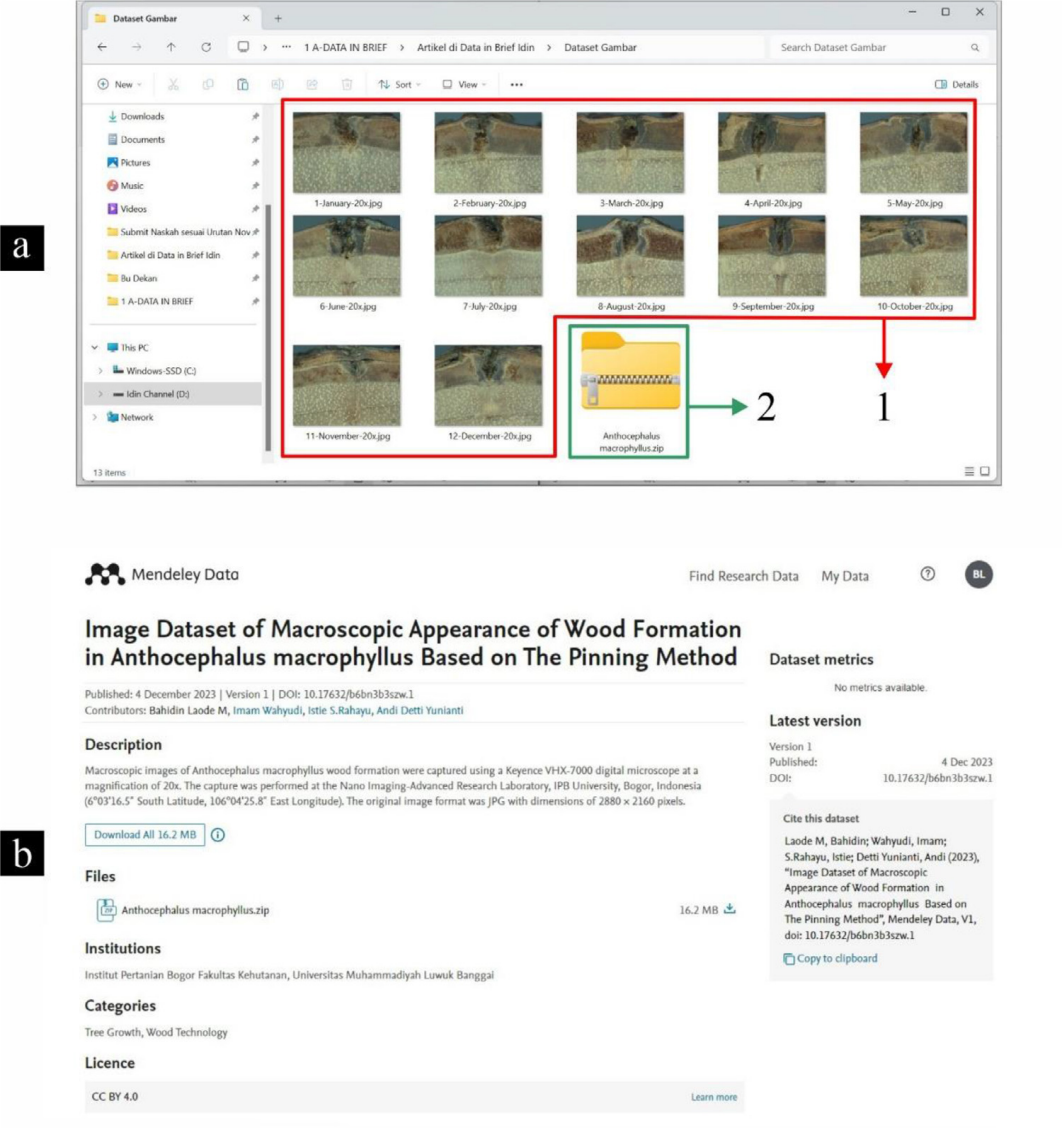
Image Dataset Management: Image folder (a): collection of images (1) and ZIP format file (2). Mendeley data repository (b).

Fig. 4 illustrates the entire image dataset obtained on 29 September 2022 at the Nano Imaging-Advanced Research Laboratory, IPB University, Bogor, Indonesia. The images were obtained in the JPG format with dimensions of 2880 × 2160 pixels. Each image file was named according to the duration of the wood formation. All images were stored in a specific folder, with a total image capacity of 16.2 MB. Subsequently, the 12 image files (January to December) were converted into ZIP format for uploading to the Mendeley Data repository [9].

It should be noted that the dataset of macroscopic images of Anthocephalus macrophyllus wood formation based on pinning is limited to only one tree species growing in a different location, soil type, and climate compared to other locations. Therefore, in the long term, we plan to expand the image dataset to include other species with different geographical coverage. The presented image dataset included only wood formation images over a one-year period, from January to December. Nevertheless, we hope that this dataset can serve as a benchmark for future research on wood formation, particularly for tropical woods.

## CRediT authorship contribution statement

**Bahidin Laode Mpapa:** Conceptualization, Data curation, Investigation, Writing – original draft. **Imam Wahyudi:** Methodology, Validation, Writing – review & editing. **Istie Sekartining Rahayu:** Methodology, Validation, Writing – review & editing. **Andi Detti Yunianti:** Validation, Writing – review & editing.

## Data Availability

Image Dataset of Macroscopic Appearance of Wood Formation in Anthocephalus macrophyllus Based on The Pinning Method (Original data) (Mendeley Data).
